# The public availability of hospital CHNA reports: limitations and potential to study hospital investments in the next phase of public health

**DOI:** 10.3389/frhs.2023.1165928

**Published:** 2023-06-08

**Authors:** Cory E. Cronin, Berkeley Franz

**Affiliations:** ^1^ College of Health Sciences and Professions, Ohio University, Athens, OH, United States; ^2^ Appalachian Institute to Advance Health Equity Science, College of Health Sciences and Professions, Ohio University, Athens, OH, United States; ^3^ Heritage College of Osteopathic Medicine, Ohio University, Athens, OH, United States

**Keywords:** hospitals, public health, community benefit, population health, data

## Abstract

Nonprofit hospitals have been required to complete and make publicly available their community benefit reports for more than a decade, a sign of changing expectations for private health care organizations to explicitly collaborate with public health departments to improve community health. Despite these important changes to practice and policy, no governmental agency provides statistics regarding compliance with this process. To better understand the nature and usefulness of the data provided through these processes, we led a research team that collected and coded Community Health Needs Assessment (CHNA) and Implementation Strategy (IS) Reports for a nationally representative sample of hospitals between 2018 and 2022. We utilized descriptive statistics to understand the frequency of noncompliance; *t*-tests and chi-square tests were employed to identify characteristics associated with incomplete documents. Approximately 95% of hospitals provided a public CHNA, and approximately 86% made their IS available. The extent of compliance with the CHNA/IS mandate indicates that these documents, paired with existing public health and policy data, offer considerable potential for understanding the investments nonprofit hospitals make to improve health outcomes and health equity in the communities they serve.

## Introduction

The Affordable Care Act (ACA) brought renewed attention and regulation to nonprofit hospitals' community benefit investments, which are required by the Internal Revenue Service (IRS) in exchange for 501(c)(3) status as charitable organizations (1). Although hospitals traditionally have met these requirements by providing free or reduced cost medical care (2) the vast expansion in health insurance coverage required new hospital activities, including investments to identify pressing community health needs, and the development of programs to improve community health. Specifically, hospitals must conduct assessments of their communities on a triennial basis, with input from with public health departments and other local health stakeholders. Hospitals must then document that they take into account the most critical community health needs when making local investments by producing public documentation in the form of community health needs assessments (CHNAs) and developing implementation strategies (ISs) to respond to community needs, which must be adopted by an authorized body of the hospital facility (3). These public CHNA reports and resulting ISs provide an opportunity for transparency by hospitals and a collaborative effort between public and private health entities within a community; they also allow for systematic analyses by public health researchers and policymakers considering whether hospitals' community-oriented investments are in response to local need, evidence-based, and of appropriate value (4, 5). A recent study found that 40% of hospitals have neglected to make these documents publicly available, and the finding raises questions about the best way to analyze these filings (6).

The aim of this report is to assess the extent of missing community benefit documents (e.g., CHNAs and ISs not made publicly available online or by request) in a nationally representative sample of hospitals, as well as to discuss strategies for using these public documents successfully in public health research. This study is significant because these reports provide considerable insight into how hospitals are making investments into their surrounding communities and very little national research exists on the availability or completeness of these reports. Hospitals are likely familiar with and attuned to the communities they are assessing, creating the potential for valuable insights both for individual communities to hold these organizations accountable and for understanding larger regional and national trends on hospital population health investments. Our study is the first to use a nationally representative dataset to assess the strengths and weaknesses of using community benefit reports, including what gaps exist in the public availability of these reports and whether they are systematic in nature.

## Methods

To better understand the role that hospitals play in improving community and population health, we established a nationally representative sample of nonprofit hospitals, and collected their CHNA and IS reports to serve as a foundation for ongoing research. Specifically, we constructed a dataset using a 20% random sample of hospitals from the national hospital population. The sample was drawn from the American Hospitals Association Annual Survey and stratified by state, to ensure that 20% of each state's hospitals were included in the sample. The characteristics of the hospitals in this sample were then compared to those in the national population and found to be representative (7).

Our next step to create the dataset was to gather CHNA and IS documents from the sampled hospitals, either by visiting hospital websites or by making direct requests to the hospitals. Because nonprofit hospitals are required to complete the CHNA process only once every 3 years, beginning in either 2012 or 2013, we used 3-year cycles (2015–2018, 2018–2021) to track each round of reports filed on a triennial basis. After downloading or receiving the publicly available PDFs, we coded these reports using a systematic protocol (8) to assess the top identified needs, whether hospitals adopted corresponding strategies to address those needs, and many additional variables related to the community benefit planning process and partnerships. If reports were not available online or were not made available by request, we coded two dichotomous variables for whether the CHNA or IS were missing. The vast majority of documents accessed for this study were posted online; hospitals without documents posted online were contacted via email with a request for their documents. Only three hospitals provided documents in response to this request.

Using the FIPS code in each hospital county, we then paired our sample of hospitals with community data from the 2018 County Health Rankings and Roadmaps Initiative and the Area Health Resource File, to assess overlap with health needs identified in secondary data, and to identify key predictors of hospitals not completing the required documentation (7, 9, 10). At the organizational level, we included hospital characteristics such as whether they were a part of a larger system (as compared to being an independent facility), the mean number of beds, whether the hospital is defined as a major teaching hospital by the American Association of Medical Colleges, and the average number of annual patient discharges. At the county level, we included measures of median income, the percent of residents who are unemployed, the percent of residents who are classified as rural, the percent of residents reporting poor/fair health (as compared to good/excellent health), the percent of residents who do not have health insurance, and the percent of residents who fall under the federal poverty line.

We calculated descriptive statistics to assess the percentage of hospitals that did not make their community benefit reports publicly available. Additionally, we used *t*-tests and chi-square tests to compare hospitals with missing reports to those that provided the reports to determine if there were systematic reasons for non-compliance. We used Stata 17 to conduct all statistical analyses.

## Results

**Figure 1:**
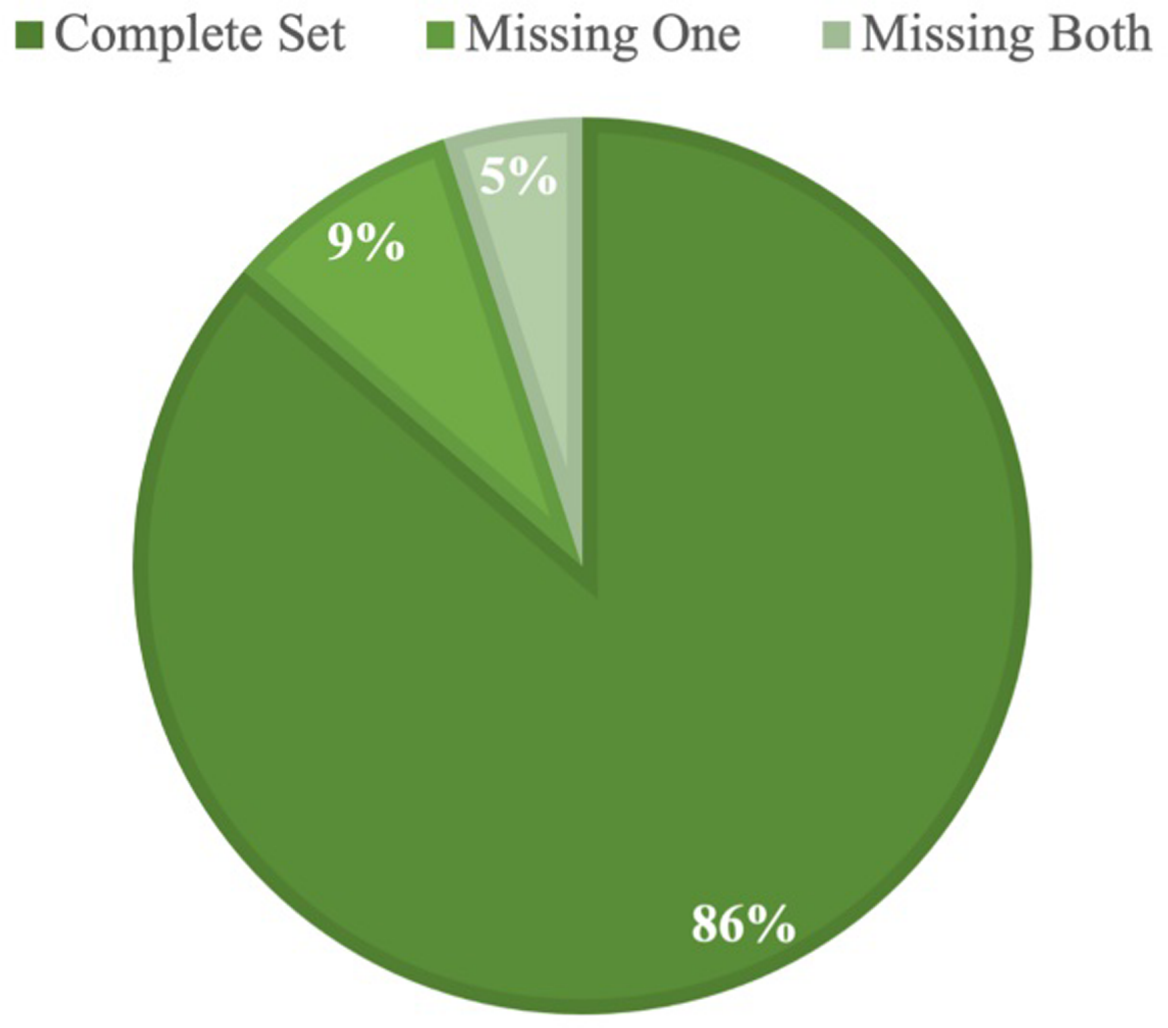
Hospital CHNA and IS completion.

In the most recent wave of data, spanning 2018–2021, we find that 503 of the 582 hospitals in our sample (86%) made both the CHNA and IS publicly available (Table 1). Of the 79 hospitals with missing reports, 29 had made neither the CHNA or IS available. We identified a 95% completion rate for CHNAs and an 86% completion rate for ISs (Figure 1). These numbers were consistent with our analysis of the previous wave of data, which showed a 94% CHNA and an 85% IS completion rate; although some hospitals that completed in the first wave did not complete in the second, and vice versa.

**Table 1:** Descriptive statistics for hospital community benefit document compliance, 2018–2021.

	Complete: publicly available CHNA and IS	Incomplete: missing CHNA and/or IS	*p*
Frequency	503	79	
Percent	86%	14%	
**Hospital characteristics**			
Hospital bed size (mean)	229	226	.921
Hospitals in health system	75%	65%	.047
Hospitals major teaching	10%	4%	.084
Hospital annual discharges (mean)	8,678	7,677	.497
**Community characteristics**			
Persons reporting poor/fair health (mean percentage)	16%	16%	.183
Uninsured (mean percentage)	9.25%	8.58%	.178
Persons in poverty (mean percentage)	13%	13%	.976
County rural residents (mean percentage)	33%	30%	.420
Unemployment (mean percentage)	3.93%	4.25%	.017
Median household income (mean)	$57,515	$56,190	.491

Overall, hospital and community characteristics appear to have little relationship to a hospital's likelihood of completing and making these reports publicly available. Only system membership and community unemployment are significant predictors of report completion. System members were significantly more likely to complete documents (*p* = .047); and hospitals in communities with higher unemployment were significantly less likely (*p* = .017).

## Discussion

Understanding the extent and pattern of missing data helps to establish these documents as reliable data for public health research and helps to identify the rate of noncompliance, which comes with consequences ranging from fines to loss of 501(c)(3) status altogether. The goal of this analysis was to understand what gaps exist in the public availability of CHNAs and ISs, and to what extent these gaps are systematic or random. By building a nationally representative dataset of nonprofit hospitals, our findings suggest that non-compliance is not as high as one recent study suggested (11). Importantly, our approach utilized the 3-year spacing required by the IRS when studying hospitals' community benefit investments, and assessing the extent of missing data which may account for this difference. Because hospitals had flexibility regarding whether to start their triennial cycle in 2012 or 2013, assessing hospital documents in any given year may overestimate the rate of noncompliance. Another factor between studies may be whether specialty hospitals such as cancer or rehabilitation centers were included in the analytic sample. Because these organizations may not have the same community health infrastructure as general community hospitals, we chose to exclude them from our sample, but inclusion of them could convey a different level of compliance across the broader hospital population.

Compliance is generally high, which strengthens the potential for these documents to be used fruitfully in research. However, looking closely at hospitals with missing data, we found that small and less capitalized institutions were less likely to complete this process, as were institutions that serve more vulnerable communities. This is potentially indicative of the role organizational resources play, as we see that hospitals able to draw on the resources of a system have higher rates of compliance. On the other hand, hospitals serving communities with economic stressors are less likely to comply, potentially due to the circumstances of their own resources. Given that less-resourced communities are ones that are likely to be most in need of close assessment and intervention, it is worth considering whether collaborative efforts across public and private sectors could provide greater support to these organizations. Direct support such as grant funding or technical assistance from local, state, or federal government sources have the potential to address gaps in resources. Organizations with fewer resources could implement such support to facilitate partnerships with public health or academic research entities or to incorporate consultants or collaboratives into the assessment process. Professional organizations, such as the Healthcare Financial Management Association, do encourage their member organizations to abide by CHNA policies; while revocation of non-profit status for noncompliance is rare, it does occur and can be financially impactful (12–14).

Going beyond compliance, and based on our experience using these data, we contend that a key issue is the broad latitude that the law grants hospitals in report quality (3, 15, 16). Greater specificity in how reports should be structured would be advantageous from a data analysis perspective. Additionally, factors such as the ability for hospital systems report at a system level rather than individual facility level dilutes the usefulness of some data; a revision to this policy would substantially enrich community-level information. Hospitals also have great latitude in deciding which community health needs to address. For example, findings from our previous studies suggest that hospitals are less willing to invest in upstream social determinants of health, as well as behavioral health needs; and face no repercussions for not responding to priority health needs identified in and by their community (8, 17, 18). For this reason, systematic analyses of the needs that hospitals identify, and those that they choose to address, are necessary. Additionally, considering whether a standardized means of collecting essential information, such as a required form in addition to a hospital's broader report, would be advantageous to public and population health efforts.

A wide range of other factors, from collaboration with public health departments and community-based institutions, to the use of consultants to produce reports, are also useful in understanding how hospitals undertake this process. Community benefit datasets have the potential to answer important questions about the scope of hospitals' population health investments, and whether current policies are sufficient to drive population health improvement. For example, explorations of broad trends in hospitals' community benefit programs have looked specifically at the program's hospitals adopt to address critical health needs such as opioid misuse and social determinants of health, while considering whether community factors (including demographic and economic) shape where hospitals make community benefit investments (11, 17, 18). Another factor worth considering is state policy regarding community benefit and, specifically, CHNA and IS documents. Hospitals that are not complying with federal policy may not be more likely to adhere to state mandates, unless such mandates come with greater enforcement. However, a more established expectation of public documents within a state may mean a hospital is already in the habit of complying with such expectations (19).

## Conclusion

As we continue to analyze the newer round of data, we intend to continue discussions on the role that nonprofit hospitals can play in improving population health and promoting health equity. Although CHNA and IS documents were not created with researchers in mind, they hold considerable potential for understanding hospital decision-making, and for holding nonprofit health care institutions accountable for community health improvement, beyond the clinical services they provide. They also offer insight into new strategies that might better drive community health improvement in collaboration with nonprofit hospitals.

For public health departments across the country that undertake their own CHNA efforts, hospital documents provide additional context and starting points for developing partnerships with health care organizations in their communities. Data gathered from these documents provide opportunities for greater collective efforts in improving population health outcomes. For this precise reason, it is worth exploring how to support those organizations that face challenges in complying with the mandated expectations in order to ensure that the needs of their communities are factored into this collective data. Greater collaboration in these efforts will continue to promote accountability and trust across public and private stakeholders within the health care sector. State health departments have the ability to play a role in incentivizing such collaborations, through the dispersal of resources.

Finally, government bodies establishing these expectations of public and private health facilities should consider the range of mandates under their purview and explore how best to align and standardize timelines and data expectations across those sectors. Private hospitals and public health departments may be more inclined to collaborate on assessment efforts and share resources and information if they have similar goals in common.

## Data Availability

The raw data supporting the conclusions of this article will be made available by the authors, without undue reservation.
